# Carbon balance analysis of agricultural production systems in oasis areas

**DOI:** 10.1038/s41598-024-66972-4

**Published:** 2024-07-19

**Authors:** Jinxiang Wang, Guohua Chang, Hao Liu, Zhuoxin Yin, Panliang Liu, Yaling Zhao, Kaiming Li, Tianpeng Gao

**Affiliations:** 1 College of Environment and Urban Construction, Lanzhou City University, The Engineering Research Center of Mining Pollution Treatment and Ecological Restoration of Gansu Province, Lanzhou, 730070 Gansu China; 2 Pratacultural College, Gansu Agricultural University, Lanzhou, 730070 Gansu China; 3 College of Biological and Environmental Engineering, Xi’an University, Xi’an, 710065 China

**Keywords:** Oasis region, Carbon emission, Agriculture production, Carbon sequestration, Environmental sciences, Environmental social sciences

## Abstract

China is the biggest emitter of greenhouse gases (GHGs) in the world, and agricultural GHG emission accounts for nearly a fifth of the total emission in China. To understand the carbon absorption and emission characteristics of agricultural production systems in those arid oasis areas, a typical representative city in northwestern China, Zhangye City, was selected for study.The emission factor method was used to analyze and calculate the characteristics of changing carbon emission dynamics in the whole agricultural production system in Zhangye city region (38,592 km^2^) from 2010 to 2021.The results revealed that carbon emissions during agricultural planting mainly come from fertilizers, which account for the highest proportion (47.9%) of total carbon emissions in agricultural planting. Animal enteric fermentation emissions from local livestock farming are the main contributor (86%) to GHG emissions. The annual average carbon absorption intensity is 4.4 t C-eq ha^−1^ for crop and 2.6 t C-eq ha^−1^ for the agricultural production system. The ratio of total carbon emissions from agricultural production to carbon sequestration of crops is 1:1.7. We find that the total carbon sequestration slightly exceeds its total carbon emissions in the study region, with an annual average of 41% for its sustainable development index. Carbon emissions of the agricultural production system in this oasis area are mainly driven by the livestock industry, mostly CH_4_ emissions from cattle raising.Reducing the local carbon emissions from the livestock industry, typically the cattle raising, will play a crucial role in reducing carbon emissions from this local agricultural production system and maintaining its net positive carbon balance.

## Introduction

The concentration of GHGs in the atmosphere has been growing continuously in the past decades, causing global warming, posing serious effects on the stability of the entire biosphere^1–3^. Climate warming has resulted in increasing frequency and intensity of climate and weather extremes, which have reduced food and water security, hindered the efforts to meet Sustainable Development Goals (high confidence). Although the global agricultural productivity has increased, climate change has slowed down this growth trend over the past 50 years (medium confidence). It is widely known that the changing climate brings a multitude of challenges, impacting everything from crop yields and livestock health to water availability and soil fertility^3,4^. In 2021 along, significant warming occurred worldwide with rising mean annual temperature averaged over global land at 1.4 °C. Nearly 170 countries and territories recorded higher than historical mean annual temperatures^5^.

In 2020, the total global GHG emissions reached 54 gigatons of CO_2_ equivalent (Gt CO_2_e) and world average per capita GHG emissions, including those from land use, land-use change and forestry emissions (LULUCF), reached 6.3 tons of CO_2_ equivalent (t CO_2_e). To get on track to limit global warming to 1.5 °C by the end of twenty-first century, global annual GHG emissions must be reduced by 45% by 2030 to avoid exhausting what remains of the finite atmospheric carbon budget^6^. Controlling and reducing carbon emissions have become one of the imperative and major challenges in the world, so as to maintain the stability of the Earth’s ecosystem in the future.

Global GHG emissions from agricultural food systems increased by 16% from 1990 to 2019, in which emissions from the agricultural food system contribute 17 billion tons of CO_2_eq, or 31% to the total global anthropogenic emissions in 2019^7^. GHGs agriculture, such as CO_2_, N_2_O, and CH_4_, are primarily emitted from soil management (e.g., fertilization, irrigation, drainage, cultivation and tillage), rice cultivation, enteric fermentation, and manure management^8–10^. Agricultural practices (planting) with reference to C emissions could be divided into primary, secondary, and tertiary sources. The primary sources (mobile or stationary operations) and secondary emission sources (fertilizers and pesticides) are important carbon emission sources in crop production operations^9,11–14^. Livestock systems are crucial in food security and livelihoods, which support the resilience of hundreds of millions of rural people across the world and contribute nearly 14.5% to the global anthropogenic GHG emissions^15^. Carbon emissions from livestock breeding, especially ruminants, are major contributors to climate change, including CH_4_ emissions that occur as part of the normal digestive process in ruminant animals, and CH_4_ and N_2_O emissions associated with manure management systems^8,16–20^.

Yet, agriculture, as a sink for GHG emissions, is unique due to its capacity to mitigate climate change via GHG emission reductions. Agricultural carbon sinks remove carbon dioxide (CO_2_) from the atmosphere through photosynthesis and carbon storage in plants and soil^3,8,21–25^. Therefore, carbon fluxes in agroecosystems entail both carbon emissions and carbon sequestration, both affecting the net carbon balance of a given agroecosystem^26^. Recently, Hu et al.^27^ systematically reviewed, summarized, and examined ecosystem carbon sequestration from 4005 articles from 1992 to 2022. They found that researches in agricultural science has mainly focused on organic carbon sequestration in farmlands. For example, the effects of tillage and organic matter addition on organic carbon sequestration in farmlands and agriculture are often linked to “nitrogen” and “forest”. However, the knowledge gaps in crop carbon sequestration by farmland ecosystems still remain and hence need more investigations. Li et al.^22^ provided a systematic summary of those peer-reviewed publications on carbon sources and sinks of farmland ecosystems in the last 30 years. These publications dealt with widely-used carbon flux computational methods and models for the carbon cycle of farmland ecosystems, and the factors influencing farmland carbon sources and sinks in the natural environment and human activities. These factors have great heterogeneity and complexity due to the differences in management practices and local natural conditions. Kay et al.^21^ examined the potentials of suitable agroforestry systems for carbon storage in European farmlands and found that their carbon sequestration potentials ranged from 0.09 to 7.29 t C ha^−1^ a^−1^, which demonstrated that agroforestry could contribute strongly to future zero-emission agriculture. Hence, maximizing the carbon sink capacity from agricultural production systems under the premise of ensuring sufficient food, feed, and other renewable resources can help extend the climate warming closing-window period and mitigate climate warming^6^.

To reduce global carbon emissions and mitigate climate change, in 2020, China put forward the goals of “striving to reach carbon peak by 2030 and carbon neutral by 2060”^28^. Since GHG emissions from agricultural production contribute 17% to China’s total emissions, reducing GHG emission from the nation’s agriculture is crucial to achieve these two goals^28,29^. Due to large disparities in natural and agricultural production conditions as well as the types of crops planted in different regions of China, the carbon emissions from different agricultural production system also differ significantly^14,29–32^. For a certain region or area, pursuing an overall analysis of planting and livestock breeding in its local agricultural production system is imperative to formulate implementable agricultural carbon-reduction strategies that are best suited to local conditions. Special concerns are raised in northwestern China. It has been reported that in the past half century, the air temperature increased at a rate of 0.034 °C per year in northwestern China, which exceeded the warming trends of China (0.025 °C per year) and the world (0.013 °C per year)^33,34^. Low precipitation (∼ 100–400 mm/year), barren lands, and scarce vegetation coverage significantly reduce the elimination of CO_2_ from the air through precipitation and forest uptake, and result in rare carbon sinks in this part of China. As a result, this region has higher CO_2_ levels than Eastern China^35^. While extensive studies have been conducted to explore and assess GHG sources and drivers contributing to GHG emissions in agricultural systems across China, little is known about these sources and drivers in arid and semi-arid northwestern China, which occupies about 30% area of mainland China. To fill the knowledge gap, the present study selected a typical agricultural region in arid Zhangye City, which is the most important agricultural area in the Heihe River Basin of northwestern China. The Heihe River Basin, the second largest inland river basin in northwestern China, has a watershed area of 12.80 × 10^6^ ha and many oases. After more than 2000 years of agricultural development, this river basin has become one of the ten key commodity grain bases in northwestern China. As a prefecture-level administrative region, it is a typical oasis city and key trade hub on the Silk Road Economic Belt, a pivotal ecological security barrier, and prominent commodity grain-planting base in Western China^36^. Taking Zhangye City as a typical case will help better understand the carbon budget characteristics of the oasis agro-ecosystem in arid northwestern China, and provide an important reference for the development of low-carbon sustainable agriculture in a fragile ecological environment with scarce vegetation coverage and carbon sinks.

## Materials and methods

### Study area

Zhangye is a prefecture level city under the jurisdiction of Gansu Province, located in the province’s western part in the northwest region of China (between 97° 20ʹ–102° 12ʹ E and 37° 28ʹ–39° 57ʹ N). Zhangye City is surrounded by Qilian Mountain to the south and Heli Mountains to the north. The city had a population of 1,122,500 at the end of 2020, and its lowest and highest elevation points are respectively 1249 m and 5542 m a.s.l. The part of the Qilian Mountain area with an elevation > 4200 m has perennial snow cover^37^. The city, covering 3.8 × 10^6^ ha, is under the temperate continental arid climate regime with dry conditions and little rainfall. Annual precipitation ranges from 104 to 328 mm, with an annual potential evaporation of 1600–2400 mm and annual sunshine duration of > 3000 h. It is cold in winter and warm in summer with an average temperature of 4–8 °C.

Zhangye City is a major agricultural base in northwestern China^38^. The main crops in the study area are spring wheat, corn, and barley. As a national modern agriculture demonstration zone and vital commodity grain base in Gansu Province, Zhangye City is also an important production base for vegetables, fruits, and oilseeds, and cattle and sheep farms^39^. The main livestock raised in Zhangye City include cows, horses, donkeys, pigs, sheep, and other animals. From 2010 to 2021, the proportion of livestock production value to total agricultural output value has increased from 16 to 32%, with an annual growth rate of 6.7% (Zhangye Statistical Yearbook (2011-2022)).

### Data and methods

According to local agriculture planting and animal husbandry practices, and corresponding data from the city’s statistical yearbook^39^, we calculated agricultural carbon emissions from agricultural production activities of agricultural planting and livestock farming, which included not only methane emissions from animal enteric fermentation but also methane and nitrous oxide emissions from animal manure management^17,40,41^. The data used here for agricultural carbon sequestration is mainly based on the available data of major local crops, such as wheat, corn, and oil crops^35^.

### Carbon emissions of agriculture planting

All data were sourced from the *Zhangye Statistical Yearbook* from 2010 to 2021^39^. Agricultural production data included crop production data, such as chemical fertilizers (net amount), pesticides, agricultural films, irrigation, tillage, and total mechanical power; the plowing area refers to the sowing area of crops. Irrigation area is the effective irrigation area.

The carbon emissions from agricultural planting production were estimated following Li et al.^41^:

1 $${C}_{\text{T}}={C}_{\text{f}}{ + C}_{\text{pf}} {+ C}_{\text{p}} {+ C}_{\text{i}} {+ C}_{\text{pt}} {+ C}_{\text{m}}=\left[\left({A}_{\text{f}}\times {F}_{\text{f}}\right)+\left({A}_{\text{pf}}\times {F}_{\text{pf}}\right)+\left({A}_{\text{p}}\times {F}_{\text{p}}\right)+\left({S}_{\text{i}}\times {F}_{\text{i}}\right)+{S}_{sa}\times \left({F}_{\text{plow}}+{F}_{\text{plant}}+{F}_{\text{harvest}}\right)+\left({W}_{\text{M}}\times {F}_{\text{M}}\right)\right]\times 0.001$$

where *C*_T_ is the total carbon emissions from the agricultural planting production (t); *C*_f_, *C*_pf_, *C*_p_, *C*_i_, *C*_pt_, and *C*_m_ are the carbon emissions (t) from the use of fertilizer, agricultural plastic film, pesticide, irrigation, mobile operations/cultivation (plowing, planting, and harvesting) and machinery, respectively (t).The *A*_f_, *A*_pf_, and* A*_p_ are the amounts of applied fertilizer, agricultural plastic film, and pesticide, respectively (kg); *F*_f_, *F*_pf_, and *F*_p_ are the carbon emission factors of fertilizer, agricultural plastic film, and pesticide, they are respective values are 0.8956, 5.18, and 4.934 kg·kg^−1^; *S*_i_ and *S*_sa_ are the effective irrigation area and sown area of crops (ha), respectively; *F*_i_, *F*_plow_, *F*_plant_, and *F*_harvest_ are the carbon emission factors of irrigation, plowing, planting, and harvest, taken as 25.00, 7.80, 6.79, and 16.47 kg ha^−1^, respectively; *W*_m_ is the total power of machinery (kW); and *F*_m_ is the carbon emission factor of machinery, taken as 0.18 kg kW^−1^^32,41,42^.

### Carbon emissions from animal husbandry

Greenhouse gas emissions from animal husbandry include methane (CH_4_) emitted from animal enteric fermentation, and CH_4_ and N_2_O emissions generated during livestock manure management. The CH_4_ and N_2_O emissions in this sector are estimated following the IPCC^17^ proposed methodology. Corresponding emission factors were taken from *Guidelines for the Preparation of Provincial Greenhouse Gas Inventories*^40^. For a livestock breeding cycle longer than 1 year, the year-end inventory of the livestock is derived by averaged annual breeding quantity. For a livestock breeding cycle shorter than one year, for example, the growth days of local pigs and poultry are respectively 200 and 55 days, the annual average of livestock population is estimated as follows^17^:

2 $$AAP = {\text{Days alive}} \times \left( {{\text{NAPA}}/{365}} \right)$$

where *AAP* is the annual mean population size; ‘Days alive’ is the number of days to raise livestock (d); NAPA is the number of animals produced annually.

The animal husbandry carbon emissions are calculated by

3 $${E}_{{\text{CH}}_{4}}=\sum ({E}_{{\text{CH}}_{4},\text{ enteric}, i}{ + E}_{{\text{CH}}_{4},\text{ manure}, i})=\sum ({{{{F}_{{\text{CH}}_{4},\text{ enteric}, i}\times P}_{i}+F}_{{\text{CH}}_{4},\text{ manure}, i}\times P}_{i})$$

4 $${E}_{{\text{N}}_{2}\text{O}}={{F}_{{\text{N}}_{2}\text{O},\text{ manure}, i}\times P}_{i}$$

5 $${E}_{\text{T}}={{E}_{{\text{CH}}_{4}}\times 7.364+E}_{{\text{N}}_{2}\text{O}}\times 74.455$$

where $${E}_{{\text{CH}}_{4}}$$ is the total amount of CH_4_ emissions from the animal enteric fermentation and manure management (kg a^−1^); $${F}_{{\text{CH}}_{4}}$$ is the emission factor of CH_4_ from the *i*-th type of animal (kg head^−1^ a^−1^), this being for enteric fermentation the mean values of large-scale farming, free-range farming by farmers, and grazing farming, while those for manure management were calculated according to emission factors for the northwest region of China (Table 1)^40^. *P*_*i*_ (head a^−1^) is the annual breeding amount of the *i*-th type of animal. $$$${E}_{{\text{N}}_{2}\text{O}}$$$$ is the total N_2_O emissions from animal manure management (kg a^−1^); $$$${F}_{{\text{N}}_{2}\text{O}}$$$$ is manure management N_2_O emission factor for the *i*-th type of animal, in units of kg head^−1^ a^−1^; and *E*_T_ is the total GHG emissions from all animals, in C_−eq_, t a^−1^^3^.

**Table 1:** GHG emission coefficients of animal enteric fermentation and manure management system of livestock (kg head^−1^ a^−1^) and the carbon conversion coefficients (*C*_i_), economic coefficients (*H*_i_), and rates of water content (*V*_i_) of different crops.

Animal type	Enteric fermentation, *F*_CH4_, enteric	Manure management, *F*_CH4_, manure	Manure management, *F*_N2O_, manure	Crop types	*C*_i_	*H*_i_	*V*_i_
Non-milk cow	60.4	1.86	0.545	Wheat	0.485	0.430	0.12
Sheep	8.45	0.28	0.074	Corn	0.471	0.438	0.13
Goat	9.15	0.32	0.074	Barley	0.450	0.510	0.12
Pig	1.0	1.38	0.195	Rice	0.410	0.450	0.12
Horse	18.0	1.09	0.33	Potato	0.423	0.667	0.70
Donkey/Mule	10.0	0.6	0.188	Cotton	0.450	0.120	0.08
				Oil plants	0.450	0.250	0.09
Camel	46	1.28	0.33	Sugar beet	0.450	0.600	0.70
Poultry	0	0.01	0.007	Vegetables	0.450	0.650	0.90

### Carbon sequestration

The carbon absorption by crops was calculated as follows^41^:

6 $${W}_{\text{crop}}=\sum {W}_{\text{i}}=\sum {C}_{\text{i}} \times {K}_{\text{i}}\times (1-{V}_{\text{i}})/{H}_{\text{i}}$$

where *W*_crop_ is the sum of carbon absorption by each crop (t); *W*_i_ is the carbon absorption by the *i*-th type of crop (t); *C*_i_ is the carbon conversion coefficient (%); *K*_i_ is the yield of the *i*-th type of crop (t); *H*_i_ is the economic coefficient of the *i*-th type of crop; and *V*_i_ is the moisture coefficient of the *i*-th type of crop. The carbon conversion coefficient, economic coefficient, and rates of water content of different crops are listed in Table 1^40,41^.

### Other carbon indicators

Other carbon indicators for agricultural ecosystems are given by

7 $$\text{Net C change }(\text{t})={W}_{\text{crop}}-\left({C}_{\text{T}}+{E}_{\text{T}}\right)$$

8 $$NEP={W}_{\text{crop}}/{S}_{\text{sa}}$$

9 $$\text{Carbon emission intensity }(\text{t}/\text{ha})=\left({C}_{\text{T}}+{E}_{\text{T}}\right)/{S}_{\text{sa}}$$

10 $$CEF=\left({C}_{\text{T}}+{E}_{\text{T}}\right)/NEP$$

11 $$I{\text{s}} = \frac{{\text{Net Cchange}}}{{W_{{{\text{crop}}}} }}$$

where *NEP* is the carbon uptake per unit of sown area (t ha^−1^)^41^; *I*s denotes the index of sustainability^9^; and *CEF* is the carbon footprint (ha) ^41,42^.

### Data analysis

MS Excel and Origin 9.0 software were used for data processing and plotting, and SPSS 21 was used for the regression analysis (using a significance level of* P* < 0.01).

## Results

### Input use of agricultural production materials

**Figure 1:**
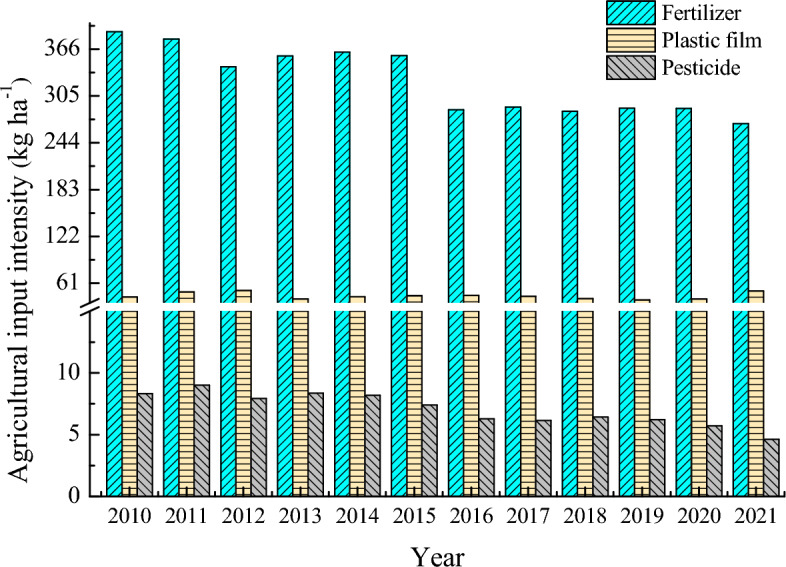
Variation in the input intensity of agricultural production materials in Zhangye City, China.

Among the three kinds of major productive resources (fertilizer, plastic film, and pesticide) invested in the agricultural production of Zhangye City, the fertilizer’s input intensity (i.e., the ratio of total fertilizer application amount to the sown area) was the greatest, followed by agricultural plastic film, and pesticide applications, respectively (Fig. 1). The fertilizer input intensity illustrates a decreasing trend over time, with the average of 324.9 kg ha^−1^. Higher intensity greater than 340 kg ha^−1^ was reported during 2010 – 2015, and subsequently dropping to 288 kg ha^−1^ from 2016 to 2020, reaching a minimum of 269.1 kg ha^−1^ in 2021.

During the 12-year period, the agricultural plastic film input intensity (i.e. the ratio between the total amount of plastic film and the sown area) ranged from 39.2 (in 2019) to 51.5 kg ha^−1^ (in 2012) with the mean at 44.4 kg ha^−1^. The pesticide input intensity also showed a declining trend with the maximum of 9.0 kg ha^−1^ in 2011 and minimum of 4.6 kg ha^−1^ in 2021.

### Carbon emissions from agricultural production

**Figure 2:**
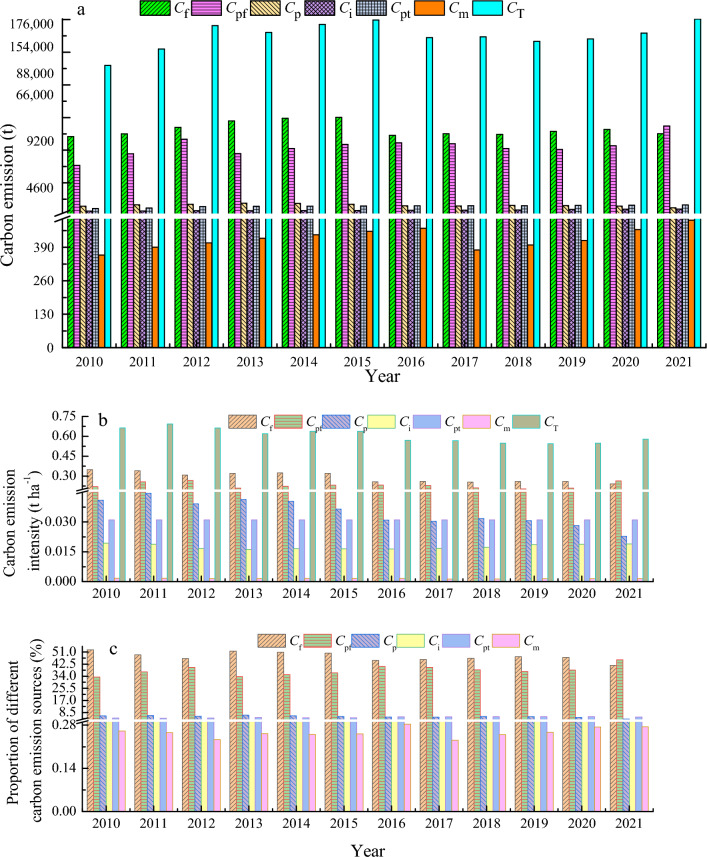
Carbon emission of agriculture from different sources (**a**), carbon emission intensity (**b**), and the proportion of different carbon emission sources from agricultural planting (**c**) in Zhangye City, China.

Figure 2 shows that carbon emissions from fertilizers (*C*_f_) in Zhangye City’s agricultural production in 2010–2015 increased on an annual basis, peaking at 90,123 t in 2015, remaining relatively stable at around 74,000–80,000 t in 2016–2021 with an annual average of 79,123 t. Carbon emissions from agricultural plastic film (*C*_pf_) were highest at 82,170 t in 2021 but below 71,000 t in other years with a rate of annual increase at 5.4% and annual average at 63,155 t during 2010–2021. From 2010–2015, carbon emissions from pesticide (*C*_p_) fluctuated, peaking at 11,223.1 t in 2013, less altered from 2016 to 2020, and being lowest at 7115 t in 2021. Both *C*_i_ and *C*_pt_ increased yearly with their respective annual increasing rates of 3.5% and 3.7%. The annual averaging values of *C*_i_ and *C*_p_ were 4835.5 t and 8549.4 t, respectively. *C*_m_ ranged from 360 to 500 t, reaching its maximum of 495.6 t in 2021. For 2010–2021, the annual averaged total carbon emissions from agricultural cultivation was 165,509.1 t, increasing by 2.5% annually and reaching the lowest value of 137,926.3 t in 2010. In the rest of years from 2010 to 2021, the total annual carbon emission were higher than 150,000 t with the maximum of 180,503.6 t in 2021.

Total carbon emission intensity (i.e. the ratio of amount of carbon emission to the sown area) from agricultural planting from 2010 to 2015 was 0.6–0.7 t ha^−1^. After that, the emission intensity declined below 0.6 t ha^−1^. The annual average of carbon emission intensity was 0.61 t ha^−1^, falling by − 1.2% annually. The carbon emission intensity in fertilizer decreased from a maximum of 0.35 t ha^−1^ in 2010 to a minimum 0.24 t ha^−1^ in 202 with the annual average was 0.29 t ha^−1^.

The carbon emission intensity in agricultural plastic film varied from 0.20 to 0.27 t ha^−1^ with an annual average of 0.23 t ha^−1^. The carbon emission intensity from pesticide dropped annually at a rate of − 5.2% a^−1^ with an annual average of 0.03 t ha^−1^. Carbon emission intensities from irrigation, cultivation, and agricultural machine were mostly constant at 0.02 t ha^−1^, 0.03 t ha^−1^, and 0.002 t ha^−1^, respectively.

In recent 12 years, the largest emission intensity was observed in fertilizer (0.29 t ha^−1^), followed by plastic film (0.23 t ha^−1^), pesticide (0.03 t/h^2^), cultivation (0.03 t ha^−1^), irrigation (0.02 t ha^−1^), and agricultural machine (0.002 t ha^−1^), respectively.

Fertilizer made the largest contribution to total carbon emissions among all sectors in agricultural planting, accounting for 42–53% of the total, but generally declined over time from the maximum of 53% in 2010 to the minimum of 42% in 2021. Emissions from plastic film ranked the second, accounting for 33–46% of the total with an annual growth rate of 2.9% a^−1^. The lowest contribution of plastic film to the total emission occurred in 2010 at 33.4%, and the maximum in 2021 at 45.5%. The contributions from other sectors were small and changed little, in which the use of pesticide contributed 5.7% to the total carbon emission, followed by cultivation (5.2%), irrigation (2.9%), and agricultural machine (0.3%).

### GHG emission variation from livestock

#### *CH*_*4*_* emissions from animal enteric fermentation*

**Figure 3:**
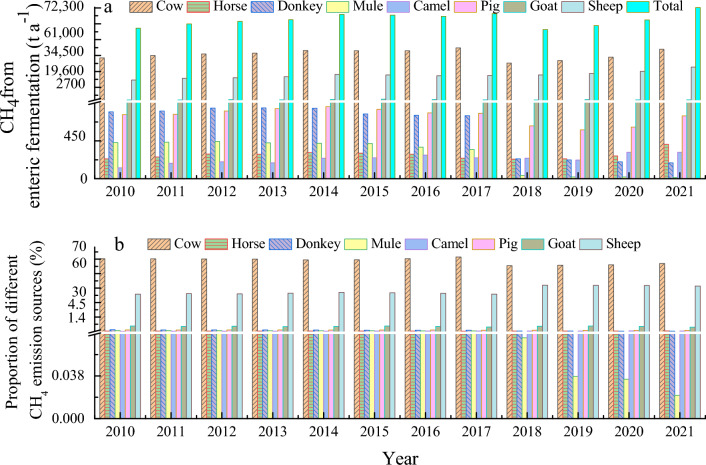
CH_4_ emissions from enteric fermentation by different animals (proportions) in Zhangye City, China.

The total CH_4_ emission from animal enteric fermentation grew slowly from 56,679 t in 2010 to 67,648 t in 2017) (Fig. 3) but decreased quickly to 55,598 t in 2018 and increased again to 72,276 t in 2021. Over the 12 years, the annual average of pooled CH_4_ emissions was 63 143 t with an annual growth rate of 2.2% a^−1^. Cattle raising dominated the CH_4_ emissions from animal sectors, showing the same trend as the total carbon emissions from all livestock pooled, reaching a maximum of 41,742 t in 2017 and a minimum of 30,357 t in 2018. The annual average of the CH_4_ emission was 37,020 t with an annual growth rate of 1.6% a^−1^. The CH_4_ emission from raising sheep also increased from 17,552 t in 2010 to 27,247 t in 2021 with an annual average of 21,308 t and growth rate of 4.1% a^−1^. The CH_4_ emission from goat varied from 2400 to 3000 t with an annual average of 2665 t and annual growth rate of 0.2% a^−1^. The CH_4_ emissions from other animals were less than 1000 t. The annually averaged emissions from horse, donkey, mule, camel, and pig were 281 t, 604 t, 283 t, 235 t, and 748 t, and their respective annual variation rates at 5.1%, − 12.2%, − 26.3%, 8.4%, and − 0.2%.

The emissions from animals’ enteric fermentation further confirmed the cattle industry dominance CH_4_ emissions, in which cattle raising contributed 55–62% to the total emission from 2010 to 2021 with an annual average of 59%. Raising sheep accounted for 31–38%, followed by goat at 4–5%. Other animals contributed less than 1% to the total in this sector.

### *CH*_*4*_* emissions from animal manure management*

**Figure 4:**
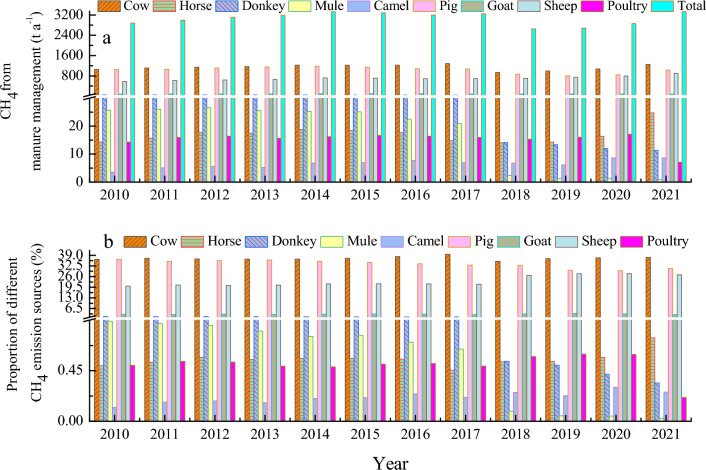
CH_4_ emissions from manure fermentation by different animals (proportions) in Zhangye City, China.

Total CH_4_ emissions from animal manure management grew slowly and ranged from 2800 to 3400 t in 2010–2017, decreased in 2018 to 2651 t and rebounded thereafter, reaching 3331 t in 2021. The annual mean CH_4_ emission averaged over 2010 to 2021 was 3064 t with an annual growth rate of 1.3% a^−1^ (Fig. 4) from 2010 to 2021.

The CH_4_ emissions released from cattle manure management show similar fluctuations to the total emissions from all farm animals with an overall increasing trend from 2010 to 2021. The maximum emission at 1286 t occurred in 2017 and the minimum was 935 t in 2018 (it was 1255 t in 2021), with an annual average of 1,140 t and an annual growth rate of 1.6% a^−1^ from 2010 to 2021.

The CH_4_ emissions from pig manure management fluctuated between 800 and 1200 t, reaching the maximum of 1182 t in 2014. When averaged over 2010 to 2021, the annual mean emission was 1033 t, having an annual growth rate of − 0.2% a^−1^. The CH_4_ emissions from sheep manure management increased on an annual basis with a minimum of 582 t in 2010 and a maximum of 903 t in 2021. The annual mean emission averaged over 2010 to 2021 was 706 t, with the annual growth rate of 4.1% a^−1^. For other animals, the annual mean emissions from their management were all less than 100 t. Among different animals, the contribution of the annual emission from cattle manure management to the total CH_4_ emission ranged from 35% in 2018 to 40% in 2017 with the annual average contribution of 37%. Pig accounted for 30–36% of the total CH_4_ emissions and annually averaged contribution of 34%, generally showing a decreasing trend. The CH_4_ emission from sheep accounted for 20–28% of the total, with an average contribution of 23%. Other animals accounted for less than 3% of the total emission from animal manure management.

### *Nitrous oxide (*N_2_O*) emissions from animal manure*

**Figure 5:**
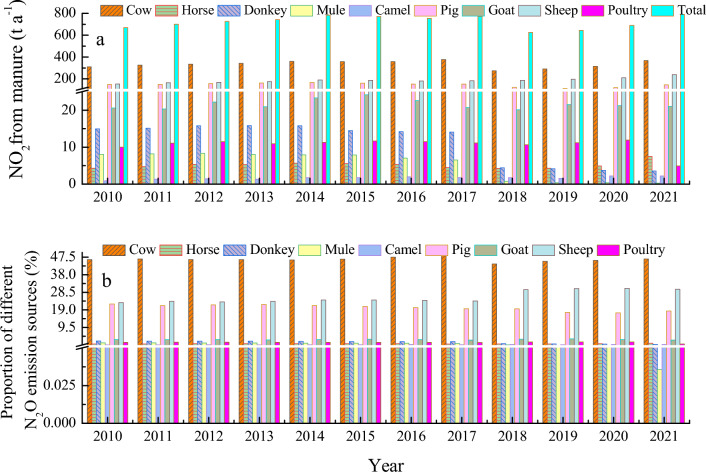
N_2_O emissions from animal manure fermentation by different animals (proportions) in Zhangye City, China.

The temporal trend of total emissions of N_2_O from animal manure management was similar to that of total CH_4_ emissions. During the 12 year-period, the minimum N_2_O emission was 626 t in 2018 and the maximum was identified in 2021 at 791. For carbon emissions, the annual average of carbon emission of 2010 through 2021 from animal manure management was 722 t with the annual rate of increase at 1.5% (Fig. 5).

The N_2_O emissions from cattle industry showed similar trends to the total emissions of N_2_O from all animals, reaching the maximum of 377 t in 2017 and the minimum of 274 t in 2018 with an annual average of 334 t and an annual increasing rate of 1.6% from 2010 to 2021. The N_2_O emission from sheep increased yearly and reached the maximum of 239 t in 2021 with an annual average 187 t, increasing annually by 4.1% during this period.

The N_2_O emissions from pig industry varied from 110 to 170 t with an annual average of 146 t and the annual rate of decline at − 0.2%. The annually averaged N_2_O emissions from other animals were less than 30 t.

Considered by animal, the proportion of emissions from cattle spanned 44% to 49%, averaging 46%. For sheep, its proportion varied around 23%–31%, with an annual average of 26%, while that of pig was 17%–22%, averaging 20%. Each of the other animals did not account for more than 3% of emissions.

We further estimated total carbon emissions by integrating all the GHG emissions from all agricultural sectors. These results showed that total carbon emissions from 2010 to 2017 increased slowly but tended to change little. The emission reached 488,471 t C-eq in 2010, 579,439 t C-eq in 2017, the minimum 475,505 t C-eq in 2018 and the maximum 615,670 t C-eq in 2021. The annual mean emission averaged from 2010 to 2021 was 541,300 t, increasing annually by 2.1%. Of that, C-eq emissions from methane enteric fermentation, methane manure management, and N_2_O manure management were 464,965, 22,560, and 53,775 t C-eq, respectively; their corresponding annual averaging growth rates were 2.2% a^−1^, 1.3% a^−1^, and 1.5% a^−1^, respectively; and contributed 86%, 4%, and 10% to the total carbon emission, respectively. As a result, the methane enteric fermentation was the main contributor to GHG emissions from local livestock in Zhangye City, followed by carbon emissions from N_2_O manure management, whereas methane manure management contributed the least.

Like for CH_4_ emissions, cattle raising’s carbon emissions dominated the total carbon emission, with an annual average of 305,864 t C-eq (an annual growth rate of 1.6% a^−1^). Carbon emissions from sheep increased yearly with an annual average of 175,999 t C-eq and an annual growth rate of 4.1% from 2010 to 2021. Overall, both cattle and sheep raising were dominant contributors to the total carbon emission at 57% and 33%, respectively, followed by pig and goat being responsible for much lower contributions of 4% each. The rest of the farm animals each only accounted for less than < 1% of the total carbon emission. Overall, the results indicate that cattle and sheep raising together accounted for 90% of the total carbon emissions. In summary, methane emissions from animal enteric fermentation made the largest contribution to the total GHG emission (86%) from 2010 to 2021, followed by the N_2_O emission from manure management (10%), and the methane emission from manure management (4%). Likewise, the ranking of carbon emissions from farmed animals was cattle (57%), then sheep (33%), and finally, the other livestock.

### Carbon sequestration in agricultural production

#### Variation in total carbon sequestration

Based on the characteristics of these locally grown crops, their planting area, and their crop yield, the carbon sequestration values from most crops are listed in Table S1. In 2010–2021, the total carbon sequestration in the region generally increased on an annual basis with an overall mean average value of 1,203,696 t C-eq for 2010 to 2021 and an annual growth rate of 2.7% a^−1^, reaching its maximum of 1,353,039 t in 2021.

Carbon sequestration of wheat was 272,589 t in 2010, which then increased slowly, peaking at 326,433 t in 2014, and decreased thereafter to 244,564 t in 2021. Annual average carbon sequestration of wheat was 277,064 t, this accounting for 23.3% of the total carbon sequestration of crops. Carbon sequestration by corn in 2010 was 499,192 t, after which it increased slowly at an annual growth rate of 4.3% y^−1^ from 2010 to 2021, reaching its maximum (795,857 t) in 2021. The annual mean values averaged over this 12 years was 659,257 t, contributing 54.5% to total carbon sequestration by crops.

Annual averaged carbon sequestrations by oil crops and barley from 2017 to 2021 were 83,078 and 68,274 t, accounting for 7.0% and 5.6% of total crop-induced carbon sequestration, respectively. The carbon sequestration of vegetables showed a trend of increasing over time, reaching their maximum (122,795 t) in 2021 with the annual growth rate of 10.6% y^−1^ and an annual average of 66,184 t, this contributing 5.4% to total carbon sequestration.

Mean carbon sequestration values for potato, rice, cotton, and sugar beet averaged over 2010 to 2021 were 38,802 t, 578 t, 1586 t, and 8873 t, respectively, collectively contributing less than 5% to total carbon sequestration. Among these crops, the carbon sequestration of sugar beet had a positive annual averaged growth rate of 8.4% a^−1^ whereas for the remaining three crops it decreased at a rate of − 0.3%, − 6.1%, and − 40.6% a^−1^, respectively, mainly due to their declining planting area and crop yield.

These carbon sequestration results in each crop type revealed that corn accounted for the greatest carbon sequestration at 55%, followed by wheat (23%), oil plants (7%), barley (6%), and vegetable (5%). The rest of the crops contributed less than 5%. As a result, corn and wheat together contributed 78% to total carbon sequestration.

#### Variation in carbon sequestration and its intensity in different crops

Based on the cultivation area of each crop, we estimated the carbon sequestration intensity for all crops (Table S2). These results showed that when pooling all crops throughout the region, their carbon sequestration intensity was 4.4 ± 0.5 t ha^−1^. Those crops having an average carbon sequestration intensity > 6 t ha^−1^ were mainly wheat, corn, cotton, and beet while for other crops it was less than < 5 t ha^−1^. Among these crops, sugar beet has the greatest carbon sequestration intensity (14.4 t ha^−1^), followed by wheat (7.1 t ha^−1^), and then corn (6.2 t ha^−1^).

Local major crops subject to large cultivation areas consisted of corn, wheat, oil crops, and potato. The carbon sequestration intensity for corn and oil crop increased annually by 2.2% and 4.2%, respectively. This suggests that the large-scale planting of corn, wheat, beet, and oil corps over large land areas across Zhangye City has advantages of increasing the carbon sink function of local farmland ecosystems.

#### Carbon indicators of the entire agricultural production system

**Figure 6:**
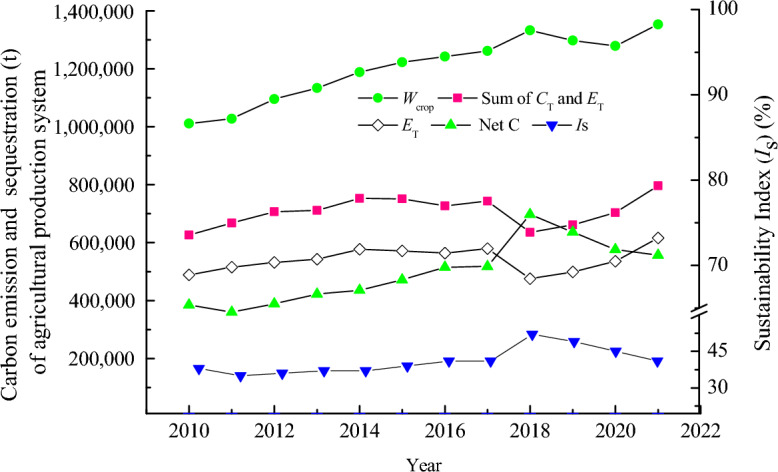
Carbon emission, carbon sequestration trend in the whole agricultural production system (left axis), and its sustainability index (*I*_S_) values (right axis) in Zhangye City, China.

The total carbon emission from local agricultural production and livestock breeding in 2010 was 626,397 t, being subsequently enhanced. In 2018, the emission amount had reached 635,417 t, but it rebounded to 796,174 t in 2021. From 2010 to 2021, the annual mean carbon emissions from agricultural planting and livestock farming were 706 810 t with an annual growth rate of 2.2% y^−1^. As a result, between 2010 and 2021, the carbon absorption of crops in the entire agricultural production system in the Zhangye City surpassed the carbon emissions from agricultural production or livestock. Accordingly, the entire local agricultural ecosystem can be considered a carbon sink. During this period, carbon absorption of the agricultural production system showed a peak, with a maximum net carbon absorption of 697,190 t in 2018 and declined thereafter. In 2021, the net carbon absorption value was 556,866 t. Overall, the annual averaged net carbon absorption of the entire system for the 12-year period was 496 886 t, for which the annual average growth rate of 3.4% (Fig. 6).

Over the past 12 years, the average carbon absorption intensity of all local crops was 4.4 t ha^−1^, of which the agricultural planting was 0.6 t ha^−1^, and animal husbandry was 2.0 t ha^−1^. Hence, the entire agricultural production system is still a carbon sink, with an average annual absorption intensity of 1.8 t ha^−1^ a^−1^. During this period, the annual average of *CEF* (Eq. 10) was 161 680 ha^−1^ with an annual average growth rate of 3.3% y^−1^. The sustainable development index of the entire system fluctuated between 35 and 52% between 2010 and 2021, peaking at 52% in 2018 but rapidly decreasing to 41% in 2021. Overall, the annual average of the sustainable development index for this period was 41%.

Over the past 12 years, the average ratio of carbon sequestration in agricultural crops to emissions from planting activities in the region was approximately 7:1. Likewise, the ratio of crop carbon sequestration to livestock carbon emissions was approximately 2:1, the ratio of agricultural planting production emissions to livestock carbon emissions was approximately 3:10; and the ratio of crop carbon sequestration to overall agricultural carbon emissions was approximately 1.7:1. The amount of carbon absorbed by local crops slightly exceeded the sum of carbon emissions from planting and livestock.

Regression analysis was conducted to further address the changes in carbon balance from carbon emissions from agricultural planting production (*C*_T_) and livestock (*E*_T_), carbon sequestration (*W*_crop_), and overall agricultural carbon emissions (Net C) over time (*t*) at *P* < 0.01. The regression equations are

12 $$C_{{\text{T}}} = {278}.{5}t^{{3}} {-}{1}.{7} \times {1}0^{{6}} t^{{2}} + {3}.{4} \times {1}0^{{9}} t{-}{2}.{3} \times {1}0^{{{12}}} ,R^{{2}} = 0.{86}0,F = {23}.{45}$$

13 $$E_{{\text{T}}} = {235}.{6}t^{{4}} {-}{1}.{9} \times {1}0^{{6}} t^{{3}} + {5}.{7} \times {1}0^{{9}} t^{2} {-}{7}.{7} \times {1}0^{{{12}}} t + {3}.{9} \times {1}0^{{{15}}} ,R^{{2}} = 0.{817},F = {7}.{8}$$

14 $$W_{{{\text{crop}}}} = {3}0{333}t{-}{6} \times {1}0^{{7}} \;R^{{2}} = 0.{916},\;F = {1}0{9}.{54}$$

15 $${\text{Net C}} = {25298}t{-}{5} \times {1}0^{{7}} R^{{2}} = 0.{717},F = {28}.{91}$$

**Figure 7:**
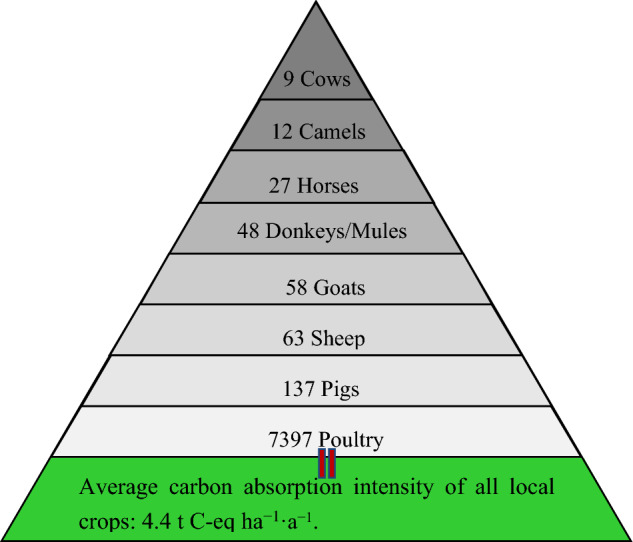
Carbon emissions subject to different animals corresponding to the average carbon absorption value of all local crops.

Based on the results of this regression analysis, it is evident that the livestock’s carbon emissions (*E*_T_) have been undergoing rapid growth. If these emissions continue to increase under most business-as-usual scenarios and are not effectively controlled, we would expect that livestock’s carbon emissions would quickly match or overwhelm the carbon sequestration of local crops in the coming years (Fig. 6). Based on the carbon calculation, the average carbon sequestration of all crops per hectare and the corresponding carbon emissions from different animals are shown in the Fig. 7. The carbon emissions generated by raising 9 cows locally in a year require planting one hectare of crops to achieve carbon balance.

## Discussion

During the 12-year period of 2010 through 2021, chemical fertilizers and agricultural films were mainly responsible for the carbon emissions generated in agricultural planting in the Zhangye City region, in which the average proportions of carbon emissions from chemical fertilizers and agricultural films accounted for 47.9% and 38.1% of their total values but the other carbon emission sources contributed < 10%. These results are consistent with Li et al.^41^, who found that carbon emissions in agriculture planting across the Qinghai-Tibet Plateau (QTP) of China mainly come from chemical fertilizers and agricultural plastic film, respectively accounting for 57% and 24% of total carbon emissions. Other carbon emissions from pesticide, machinery, and irrigation in the QTP region were all below 10% with the carbon emissions per unit of cultivated area from chemical fertilizers at 357.80 kg ha^−1^ in 2015. Chemical fertilizer also accounted for the largest proportion of GHG emissions in raw material from the agricultural production system in the Shaanxi Province of China^43^. Fertilizer application is one of the main contributors to the production carbon footprint of staple grains in China and GHG emissions from agricultural productions^14^.

To meet the rising food demand of a growing world population, synthetic nitrogen (N) fertilizer has been widely used to improve biomass production^13^. The synthetic N fertilizer supply chains entail significant GHG emissions. The both can sourced from their production and from their actual use. According to FAO estimates^5,12^, total global agricultural consumption of elemental N from synthetic fertilizers reached 107.7 Mt in 2018, contributing to 1129.1 ± 171.1 Mt CO_2_e (mean ± sd) GHG emissions. This value includes the GHGs emitted by fertilizer manufacturing and transportation, and the subsequent direct and indirect soil emissions resulting from fertilizer application on agricultural lands^12^. When N fertilizer is applied to soil, only a small portion of it is actually absorbed by plants, and the rest of it may be transformed into N_2_O under soil microbial activities that may end up via leaching into or volatilizing from an application site. The FAO has reported that global annual N_2_O emissions from synthetic fertilizers increased by 44% between 1990 and 2019 and predicted that worldwide use of synthetic N fertilizers will increase 50% from 2012 to 2050, which, in turn, will potentially jeopardize the realization of the climate objective of the Paris Agreement’s 1.5 °C and 2 °C targets^12^. Data from FAO also show that the agricultural consumption of elemental N from synthetic fertilizers used in China reached 28.1 Mt, which released 316.1 ± 113.3 Mt CO_2_e in 2018^12^, indicating that China has become the largest emitter globally. Hence, reducing the overall production and use of synthetic N fertilizers and over-fertilization application can help to minimize GHG emissions and their global warming potential. The mean annual chemical fertilizer input intensity in Zhangye City was more than 250 kg ha^−1^. Such strong intensity was primarily attributed to China’s low price regulation on fertilizer industries and the bounty to peasant households. While the low prices of fertilizers can help farmers to reduce their expenses in grain cultivation, the cheap fertilizers result in excessive dependence on fertilizer for agricultural production. Further, the artificially low production cost of fertilizer weakens the competitiveness of substitutable products. For these reasons, there is general overuse of fertilizer in the study area. According to statistical data of China’s agricultural sector, the application of nitrogen fertilizer had been growing slowly since 2000, having peaked at 23.999 million tons in 2012 but fallen since to 17.453 million tons in 2021^44,45^. However, according to the statistics of China's agricultural sector, China’s nitrogen fertilizer application has gradually declined since 2000, reaching a peak of 60.226 million tons in 2015. The decline of fertilizer application is closely related to the fact that the Chinese government has formulated and actively implemented its ‘Action Plan for Zero Growth of Fertilizer Use by 2020’ throughout the country since 2015, aiming to control and reduce the use of chemical fertilizers in agriculture, protect water resources, and minimize GHG emissions^46^. Given large differences in resource endowment, agricultural modernization level and structure, the population, economy, and technologies, there are stark regional differences in the activities of agricultural carbon emission reductions across China^31^. The intensity of agricultural carbon emission sources is relatively stronger in those provinces of Western China than those in Central and Eastern China^29^. To uncover and attain maximal “win–win” outcomes between crop yield and GHG emission intensity, Guo et al.^47^ conducted a global meta-analysis of crop yields and agricultural GHG emissions subject to nitrogen fertilizer application. They found that nitrogen fertilization is primarily suitable for arid and semi-arid areas featured by medium and neutral or alkaline soils, for which the recommended N fertilization rates are no more than 200 kg ha^−1^ for crops, which could not only maximize crop yield, but also not exceed the global warming potential. Since 1986, China has invested nearly 700 million Yuan in the research and development of eco-agricultural technology. The progresses in agricultural technology have played a positive role in reducing agricultural carbon emissions in different regions of China, especially in Eastern China^32^.

The livestock sector contributes approximately 14.5% of GHG emissions, of which the enteric fermentation is the largest contributor to GHG emission in the animal production stage^19,48^. Ruminant animals are responsible for 75% of the total carbon dioxide equivalent (CO_2_-eq) emissions from the livestock sector with bovines comprising the bulk of these emissions^15^. In China, the consumption of ruminant meat and dairy products has increased exponentially from the early 1990s and 2000s, respectively, and the total demand for ruminant products in China is predicted to double by 2050. The latter has become a fundamental driving force for increasing CH_4_ emissions^49^. For example, the beef consumption in Chinese households increased from 1.5 kg per capita in 2013 to 2.3 kg per capita in 2020^45,50^.

From 1997 to 2016, the agricultural carbon emissions in China exhibited an increasing trend and reached 373.9123 million tons in 2016, rising by 19.8% with an average annual growth rate of 0.959% ^29^. Agricultural non-CO_2_ GHG emissions enhanced by 34% from 1980 to 2018 in China, and they are projected to increase further by 33% to reach 1153 MtCO_2_-eq yr^−1^ by 2060^30^. The non-CO_2_ GHG emissions from 2018 to 2060 in Gansu Province will rise by more than 40% under the ‘business-as-usual’ scenario, attributable mainly to the rapid growth of ruminants and enteric fermentation^30^. The median total CH_4_ emitted from the livestock sector in China in 2014 was 10.8 Tg CH_4_·yr^−1^, of which the largest CH_4_ emission source is beef cattle at 2.8 Tg CH_4_·yr^−1^^51^. In 2015, the carbon emission from animal husbandry over the QTP in China amounted to 87 157,614 CO_2_-eq t, in which cattle rising made the larest contribution of 80% to the total emissions^41^.

Agriculture is both a source and a sink of GHGs. In the case of the U.S., its agricultural sector is a net GHG source. According to the US-EPA reports of GHG estimates as CO_2_-equivalents, that agriculture sector’s emissions totaled 635.1 millions of metric tons (MMTCO_2_e) in 2020; among them, enteric fermentation (28%, 175.2 MMTCO_2_e) and manure management (13%, 79.2 MMTCO_2_e) accounted for 11% of total U.S. GHG emissions (5981.4 MMTCO_2_e)^8^. Argentina is one of the main global producers of beef, accounting for about 5% of global production, and in 2016 its livestock sector emitted 157 Mt CO_2_-eq into the atmosphere (46 Mt CO_2_-eq from land-use change). Yet the relative weight of each livestock group in determining the total environmental footprint differed, with beef being the dominant one. Delivering protein from beef requires between 12 and 28 times more land, and emits 6–34 times more GHGs than does all other livestock products such as pork and chicken^52^.

The average total carbon emission from agricultural cultivation and livestock in the Zhangye City region over the past 12 years was 706,810 t, of which 77% came from livestock. Of the annual total emissions of local livestock, the proportion of animal enteric fermentation is as high as 86%, and hence the main contributor to GHG emissions from the local livestock farming industry. The cattle industry was the largest emitter, accounting for 57% of the total, followed by sheep (33%). Therefore, the carbon emissions from local livestock, especially from cattle, are the main cause of carbon emissions in the local agricultural system. According to a Zhangye City’s administrative report, Ganzhou District and Linze County of Zhangye City had 261,300 and 143,700 head of cattle, accounting for 39% and 21% of the region’s total, respectively, cattle from other areas had no more than 15% of the total in the Zhangye City region in 2021^39^. Hence, more control measures should be implemented in these two areas, including scientifically and reasonably planning the feeding scale of different livestock, optimizing the livestock feeding structure, implementing cooperative crop livestock systems, and improving both feed efficiency and manure management for reducing GHG emissions from animal husbandry. Recent research by Xu et al.^51^ and Ma et al.^53^ indicated that adding lipid to diets and the combination of composting and anaerobic digestion were the most effective CH_4_ mitigation technologies. Their results reveal that, compared to decoupled specialized livestock systems (DSLS), the cooperative crop-livestock system (CCLS) could yield lower net GHG emissions (12%–29%), lower reactive nitrogen emissions (21%–40%), lower phosphorus footprints (41%–54%), and used less cropland (24%–31%) per kg animal product. Cooperation between specialized livestock and crop farms can reduce the use of synthetic fertilizer by augmenting the recycling and use of animal manure, which are main aims of “agriculture green development” in China^53^.

Using a major Australian irrigated agriculture system as a case study, Maraseni et al.^54^ developed an integrated water–food–energy nexus optimization model and found that optimizing the use of land and water resources can achieve greater crop yields and reduce GHG emissions, which also help sustain economic outcomes. At a regional scale, up to a 50% reduction in GHG emissions from irrigated crop production is possible without compromising total gross margins. The regional optimization of resource use could also result in surplus water and land available for environmental planting.

From 2010 to 2021, the total carbon absorption of all crops in Zhangye City region showed a slow annual growth trend with the annual growth rate 2.7%. In 2021, the total carbon absorption increased to its maximum value of 1,353,039 t in which corn contributed most to carbon absorption, account for 55%, followed by wheat (23%). The average annual carbon absorption intensity of all crops in the entire region fluctuated between 4.4 ± 0.5 t ha^−1^, closely matching the carbon sequestration per unit area of the QTP^41^. The quantification of combined GHG budgets for pea and maize revealed that CO_2_ uptake offsets N_2_O and CH_4_ emissions, and both crops act as GHG sinks during the cropping season with the (absolute) net CO_2_ uptake of pea and maize being 514 and 1944 g CO_2_ m^−2^, respectively^23^.

Crop carbon sequestration plays an undeniable role in reducing carbon emissions. Carbon sequestration strategies can be broadly classified into two categories: biological and non-biological. When compared with non-biological methods, biotic techniques are cost-effective and immediately applicable. Photosynthetic uptake of CO_2_ is primarily responsible for global carbon cycling. About 9 gigatons (Gt) of carbon is released annually into the atmosphere by human activities, of which 5 Gt is naturally absorbed by the aquatic and terrestrial systems^37^. Recently, Kuyah et al.^55^ reviewed the contributions of grain legumes and dryland cereals to carbon sequestration across the drylands of Africa and South Asia, based on 437 publications with 1319 observations across 32 countries. They found that cropping systems with grain legumes led to the greatest increase in soil organic carbon (SOC) concentrations, while farming cereals (and pigeon pea) led to the largest aboveground carbon stock (> 2 Mg C ha^−1^). The estimated carbon stock in post-harvest residues of these crops was 1.51 ± 0.05 Mg C ha^−1^ in Africa and 2.29 ± 0.10 Mg C ha^−1^ in South Asia. These crops not only produced more aboveground carbon but also significantly increased SOC when growing as intercrops. Agroforestry is widely recognized as a promising land use strategy that delivers benefits for climate change adaptation and mitigation, including synergies with climate change mitigation via carbon sequestration, enhanced food security and income opportunities, the provisioning of ecosystem services, and biodiversity conservation^56,57^. Achievement of 8.9% agroforestry across European agricultural lands could potentially reduce the total European agricultural GHG emissions by 1.4–43.4% of t, which is encouraging and demonstrates that agroforestry could contribute strongly to preparing the ground for a future world of zero-emission agriculture^21^. Agroforestry can also play a major role in reaching national, European, and global climate targets, and simultaneously fostering environmental policy and promoting sustainable agriculture, particularly in those areas with intensive agricultural management and large environmental pressures^21^.

Oasis ecosystems are extremely valuable natural resources in arid areas and critical landscapes in the human environment system. Located in an oasis area with a fragile and sensitive ecological environment spanning semi-arid and arid regions, Zhangye underpins the production and livelihood of millions of people, and is a prominent node of the Silk Road Economic Belt^37^. Safeguarding the ecological environment of oases, especially their water resources, land resources, and surface vegetation systems and maintaining its carbon balance, is paramount for ensuring the stable development of oasis ecosystems and achieving sustainable ecological, economic, and social development. The Central government of China has set up special support for the standardized transformation of large-scale livestock farms by constructing supporting facilities for manure treatment, such as septic tanks and sewage discharge pipelines, and by reducing GHG emissions from animal husbandry. In 2015, China implemented a series of actions such as vigorously developing water-saving agriculture, pursuing zero-growth action for fertilizers and pesticides, promoting pollution prevention and control in aquaculture, and deepening the utilization of straw resources^58^. In recent years, the Zhangye Municipal Government has adhered to those policies and continues to carry out the reduction of fertilizers, pesticides, and plastic film, and is building demonstration sites for the recycling of farmland residual film. In 2021, the recycling and utilization rate of waste agricultural film in the city was 86.92%, the comprehensive utilization rate of straw was 90.96%, and the fertilizer and pesticide utilization rates were 40.92% and 41%, respectively^59^.

Sustainable development of the local agricultural production system as a whole still requires further improvement. In this study, we did not analyze the carbon budget from each administrative divisions of the city, and the statistical time span is relatively short. Additional in-depth analyses are needed in future studies, especially in those areas with higher levels of cattle farming. Further optimization of livestock farming techniques and conditions should be carried out to reduce GHG emissions. Efforts should be also made to scientifically and reasonably plan the scale of farming different livestock, so as to optimize the structure of livestock farming to reduce GHG emissions. The application of integrated digital technologies to pave the way forward for future smart agricultural systems provides promising possibilities for bolstering the competitiveness, sustainability, and resilience of the agricultural sector, as well as reducing its carbon emissions^60^.

Further efforts should be also made in developing smart agriculture, optimize land and water use, promoting the application of modern agricultural technology, saving precious water, increasing agricultural output value, implementing complementary agricultural and forestry systems, and reducing GHG emissions from agricultural systems, especially from animal husbandry. This is also a pivotal direction for protecting the ecological environment of oases and promoting the sustainable development of oasis agriculture more broadly.

## Conclusions

The reduction of carbon emissions of oasis agricultural systems in arid areas has a significant impact on protecting the local ecological environment and realizing sustainable agricultural development. The present study shows that the entire agricultural production system in Zhangye City acts as a carbon source. The use of fertilizers is the main driver of the carbon emissions in the agricultural planting industry, accounting for almost half of its total carbon emissions. Carbon emission from cattle in the livestock industry is the main source of carbon emissions in the local agricultural system. The ratio of crop carbon sequestration to overall agricultural carbon emissions is approximately 1.7:1. Hence, the overall agricultural ecosystem acts as a carbon sink. The total annually averaged net carbon emission of the entire system during the recent 12-year period (2010–2021) was 496,886 t with an annual growth rate of 3.4% but the system carbon absorption intensity in the entire system is negative. Therefore, reducing carbon emissions from fertilizers and livestock, especially cattle, will play an instrumental role in maintaining the local carbon balance. To achieve this goal, efforts need to be made to promote agricultural technological innovation, improve the efficiency of agricultural resource utilization, develop environment friendly fertilizers, and reduce the utilization rate of fertilizers. To achieve more sustainable animal husbandry industry, it is recommended to improve breeding and feeding methods, scientifically manage animal manure, and adhere to a combination of moderate breeding scale and scientific utilization of waste.

### Supplementary Information

Supplementary Tables.

## Data Availability

The datasets used and analyzed during the current study are available from the corresponding author on reasonable request.
