# Supplementary material for: Carbon balance analysis of agricultural production systems in oasis areas
Source: Sci Rep. 2024 Jul 19;14:16698. doi: 10.1038/s41598-024-66972-4 (PMC11271539; doi:10.1038/s41598-024-66972-4)
Supplement: Supplementary file 1 — Supplementary Tables. [file 41598_2024_66972_MOESM1_ESM.docx]

Supplementary Material for

**Title:** **Carbon balance analysis of agricultural production systems in oasis areas**

**Authors**: Jinxiang Wang^a^, Guohua Chang^a,*^, Hao Liu^b^, Zhuoxin Yin^a^, Panliang Liu^a^, Yaling Zhao^a^, Kaiming Li^a^, Tianpeng Gao^a, c,*^

**Affiliation**:

^a^*College of Environment and Urban Construction, Lanzhou City University, The Engineering Research Center of Mining Pollution Treatment and Ecological Restoration of Gansu Province, Gansu 730070, China*

^b^*Pratacultural College, Gansu Agricultural University, Gansu 730070, China*

^c^*College of Biological and Environmental Engineering, Xi'an University, Xi'an 710065, China*

***Corresponding author**: *Guohua Chang, [cgh@lzcu.edu.cn](mailto:cgh@lzcu.edu.cn); [494164311@qq.com](mailto:494164311@qq.com)

The word is 4 pages in total, including Table S1 and S2.

Supplementary Material

**Table S1**

Amount of carbon sequestration by different crops and their proportions in Zhangye City, China.

**Table S2**

The carbon sequestration intensity (t ha^−1^) of different crops in Zhangye City, China.

**Table S1**

Amount of carbon sequestration by different crops and their proportions in Zhangye City, China.

| Year | Wheat (t) | | Corn (t) | | Barley (t) | | Rice (t) | | Potato (t) | | Cotton (t) | | Oil Plants (t) | | Sugar beet (t) | | Vegetable (t) | |
| --- | --- | --- | --- | --- | --- | --- | --- | --- | --- | --- | --- | --- | --- | --- | --- | --- | --- | --- |
| 2010 | 272589 | 27.0% | 499192 | 49.4% | 54612 | 5.4% | 1347 | 0.1% | 37107 | 3.7% | 2997 | 0.3% | 96626 | 9.6% | 6028 | 0.6% | 40523 | 4.0% |
| 2011 | 271820 | 26.4% | 537320 | 52.3% | 47331 | 4.6% | 1215 | 0.1% | 42279 | 4.1% | 3898 | 0.4% | 72982 | 7.1% | 7649 | 0.7% | 43385 | 4.2% |
| 2012 | 286452 | 26.2% | 564973 | 51.6% | 58581 | 5.3% | 220 | 0.0% | 45212 | 4.1% | 3950 | 0.4% | 81047 | 7.4% | 8702 | 0.8% | 45961 | 4.2% |
| 2013 | 303036 | 26.7% | 590778 | 52.1% | 52374 | 4.6% | 384 | 0.0% | 45640 | 4.0% | 3612 | 0.3% | 78015 | 6.9% | 5460 | 0.5% | 54403 | 4.8% |
| 2014 | 326433 | 27.5% | 600235 | 50.5% | 57507 | 4.8% | 502 | 0.0% | 44769 | 3.8% | 2864 | 0.2% | 87301 | 7.3% | 8968 | 0.8% | 59514 | 5.0% |
| 2015 | 306581 | 25.1% | 656504 | 53.7% | 55660 | 4.6% | 173 | 0.0% | 42426 | 3.5% | 934 | 0.1% | 92126 | 7.5% | 5228 | 0.4% | 63012 | 5.2% |
| 2016 | 293412 | 23.6% | 692882 | 55.8% | 57498 | 4.6% | 316 | 0.0% | 43012 | 3.5% | 257 | 0.0% | 92787 | 7.5% | 6297 | 0.5% | 55571 | 4.5% |
| 2017 | 279850 | 22.2% | 700097 | 55.5% | 68518 | 5.4% | 429 | 0.0% | 38237 | 3.0% | 28 | 0.0% | 95540 | 7.6% | 14806 | 1.2% | 63922 | 5.1% |
| 2018 | 249693 | 18.7% | 768228 | 57.6% | 83502 | 6.3% | 148 | 0.0% | 29632 | 2.2% | 317 | 0.0% | 118714 | 8.9% | 8896 | 0.7% | 73476 | 5.5% |
| 2019 | 251115 | 19.3% | 750748 | 57.8% | 96288 | 7.4% | 308 | 0.0% | 28487 | 2.2% | 161 | 0.0% | 82671 | 6.4% | 7861 | 0.6% | 80175 | 6.2% |
| 2020 | 239223 | 18.7% | 754265 | 59.0% | 98682 | 7.7% | 1218 | 0.1% | 33108 | 2.6% | 7 | 0.0% | 49016 | 3.8% | 12010 | 0.9% | 91472 | 7.2% |
| 2021 | 244564 | 18.1% | 795857 | 58.8% | 88736 | 6.6% | 675 | 0.0% | 35714 | 2.6% | 10 | 0.0% | 50116 | 3.7% | 14572 | 1.1% | 122795 | 9.1% |

**Table S2**

The carbon sequestration intensity (t ha^−1^) of different crops in Zhangye City, China.

| Year | Wheat | Corn | Barley | Rice | Potato | Cotton | Oil Plants | Sugar beet | Vegetable | Total |
| --- | --- | --- | --- | --- | --- | --- | --- | --- | --- | --- |
| 2010 | 7.4 | 5.8 | 4.8 | 3.4 | 1.4 | 6.2 | 3.3 | 20.1 | 3.8 | 4.9 |
| 2011 | 7.4 | 5.9 | 3.1 | 3.6 | 1.5 | 6.0 | 2.2 | 13.8 | 3.8 | 4.7 |
| 2012 | 7.2 | 5.4 | 3.9 | 5.5 | 1.6 | 6.4 | 3.2 | 17.2 | 3.6 | 4.2 |
| 2013 | 7.4 | 5.2 | 3.8 | 3.4 | 1.6 | 6.7 | 3.6 | 16.7 | 3.6 | 4.2 |
| 2014 | 7.8 | 5.6 | 4.0 | 3.3 | 1.7 | 6.6 | 2.9 | 15.6 | 3.5 | 4.3 |
| 2015 | 7.7 | 5.7 | 4.1 | 2.6 | 1.6 | 7.0 | 2.9 | 13.1 | 3.7 | 4.3 |
| 2016 | 7.7 | 5.7 | 4.2 | 3.0 | 1.7 | 7.7 | 2.9 | 12.6 | 3.4 | 4.3 |
| 2017 | 7.8 | 5.7 | 4.3 | 3.2 | 1.9 | 4.1 | 3.1 | 13.6 | 3.5 | 4.4 |
| 2018 | 5.9 | 7.0 | 4.3 | 7.4 | 1.4 | - | 3.9 | 14.5 | 3.8 | 4.6 |
| 2019 | 6.1 | 7.2 | 4.1 | 6.6 | 1.4 | - | 3.6 | 11.7 | 3.2 | 4.4 |
| 2020 | 6.3 | 7.2 | 4.5 | 6.3 | 1.5 | - | 4.5 | 11.5 | 3.3 | 4.2 |
| 2021 | 6.5 | 7.4 | 4.6 | 7.2 | 1.5 | - | 5.3 | 12.9 | 3.3 | 4.3 |
| **Average** | **7.1** | **6.2** | **4.1** | **4.6** | **1.6** | **6.3** | **3.4** | **14.4** | **3.5** | **4.4** |
